# From Classical to Unconventional: The Immune Receptors Facilitating Platelet Responses to Infection and Inflammation

**DOI:** 10.3390/biology9100343

**Published:** 2020-10-20

**Authors:** Iluja Gautam, Zachary Storad, Louis Filipiak, Chadwick Huss, Claire K. Meikle, Randall G. Worth, Leah M. Wuescher

**Affiliations:** Department of Medical Microbiology and Immunology, University of Toledo College of Medicine and Life Sciences, Toledo, OH 43614, USA; iluja.gautam@rockets.utoledo.edu (I.G.); zachary.storad@rockets.utoledo.edu (Z.S.); louis.filipiak@rockets.utoledo.edu (L.F.); Chadwick.huss@rockets.utoledo.edu (C.H.); Claire.meikle@rockets.utoledo.edu (C.K.M.); randall.worth@utoledo.edu (R.G.W.)

**Keywords:** platelets, PRRs, integrins, complement, viruses, bacteria

## Abstract

**Simple Summary:**

Platelets are small and numerous cells, second only to red blood cells in the bloodstream. They have been recognized mainly for their contributions to blood clotting, however, we now know that they also play important roles in our responses to infection and inflammation. In this extensive review, we cover the “classic” platelet receptors involved in blood clotting and “unconventional” immune receptors that platelets possess and how both groups contribute to our immune responses. Platelet receptors can interact with bacteria and affect the behaviors of white blood cells which fight infection. Additionally, these receptors can be involved in autoimmune conditions, when the body mounts an immune response against itself. Continued research on these interactions could lead to development of new treatments against numerous diseases.

**Abstract:**

Platelets have long been recognized for their role in maintaining the balance between hemostasis and thrombosis. While their contributions to blood clotting have been well established, it has been increasingly evident that their roles extend to both innate and adaptive immune functions during infection and inflammation. In this comprehensive review, we describe the various ways in which platelets interact with different microbes and elicit immune responses either directly, or through modulation of leukocyte behaviors.

## 1. Introduction

The normal hemostatic function or “classical activation” of platelets has been well established (reviewed in [1,2]). Briefly, we will examine the classic platelet receptors and how they contribute to hemostasis, the cessation of bleeding. Upon endothelial injury, extracellular matrix (ECM) proteins such as von Willebrand factor (vWF) and collagen are exposed to the blood. vWF can accumulate at the site of injury and, in turn, platelet GP1bα (part of the GP1b-V-IX complex, vWF receptor) can bind to the surface. Platelets then slow and roll over the ECM proteins, causing engagement of the collagen receptors α2β1 and GPVI. Ligation of these receptors leads to signaling processes that activate integrins such as αIIbβ3 and cause granule release, both necessary for platelet plug formation (primary hemostasis).

Platelets possess 3 distinct granule types: alpha (α), dense (δ), and lysosomal, with α- and δ- granules playing important roles in hemostasis [3]. Alpha granules possess a wide array of molecules ranging from soluble mediators such as fibrinogen, coagulation factors, chemokines, and microbicidal proteins to adhesion proteins such as P-selectin. Dense granules contain the bioactive amines (e.g., serotonin, histamine), polyphosphates, ADP, ATP, and cations (e.g., calcium). Notably, platelet activation also leads to generation of thromboxane A2, which, along with many of the mediators mentioned above, is integral in the formation of platelet aggregates through activation of its G-protein coupled receptor (GPCR). The thromboxane receptor (TP) and the ADP receptor (P2Y_12_) are critical for amplification of platelet signaling and aggregate formation. 

Clot stabilization is achieved through generation of a fibrin lattice around the platelet plug (secondary hemostasis). Activation of the coagulation cascade through both recognition of endothelial damage and platelet activation leads to generation of the protease thrombin. Along with being able to cleave fibrinogen to fibrin, it is also a potent activator of platelets through cleavage of the N-terminal domain of the protease activated receptors (PARs). This further contributes to the feed forward mechanism of platelet activation leading to stable clot formation [2]. 

The role of the platelet in processes other than clot formation has become more expanded and understood in recent years. For example, platelets are now appreciated for their role in immunity due to the characterization of functional immune receptors intracellularly and on their surface [2]. While mammals have evolved separate systems for immunity and coagulation, looking at evolutionary examples of organisms which have one cell type responsible for both coagulation and immunity could give insight as to why platelets express these receptors.

Invertebrates most commonly have a solution called hemolymph cycling through their open circulatory systems to the hemocoel, which is a cavity where hemolymph bathes organs with nutrients. Hemolymph is the invertebrate equivalent of blood, but with no distinction of interstitial fluid and blood cells [4]. It is also where hemocytes (invertebrate blood cells) reside. Hemocytes include different subsets or classifications, but many can respond when exposed to pathogens. For instance, in the horseshoe crab, granular hemocytes have a robust response to lipopolysaccharide(LPS), releasing their granular contents including microbicidal proteins such as tachyplesin, tachystatins, big defensin, and tachycitin [5]. In addition to harboring microbicidal proteins, hemocytes also contain procoagulant molecules vital to tissue repair [6]. However, similar to vertebrates [7], these factors can also be activated in the presence of microbes to form a physical barrier and sequester microbes at the site of infection [5]. 

Higher up the evolutionary chain, small nucleated thrombocytes with features similar to that of the mammalian platelet are present in species such as birds, reptiles, fish, and amphibians [8,9]. Not only do these nucleated thrombocytes play a role in coagulation [10,11], but, similar to mammalian platelets, have been shown to play significant roles in the immune responses of these organisms [9,12,13]. For example, avian thrombocytes and fish thrombocytes have been reported to possess functional toll like receptors that initiate immune responses to various pathogens, in addition to being able to phagocytose beads, viruses, and bacteria [12,13]. 

The evolutionary evidence we have available begins to suggest why platelets possess these types of receptors. However, not only do platelets have pattern recognition receptors (PRRs), they also express distinct receptors important in facilitating immune responses. In this review, we will examine the classical and unconventional receptors expressed by platelets and their important roles in immunity.

## 2. Adhesion Receptors in Platelets Are Crucial for Platelet Aggregation and Participate in Different Infections

Adhesion receptors are membrane proteins which allow for cell-cell interactions as well as cell-extracellular matrix interactions [14]. In addition to their crucial role in developmental processes, these receptors are important in immunity. Based on their structure, adhesion receptors are classified into four families: integrins, cadherins, selectins, and intercellular adhesion molecules (ICAMs) [15]. 

### 2.1. αIIbβ3 Is Involved in Pathogen Recognition and Molecular Mimicry

Integrins are a large family of transmembrane glycoproteins (GP) comprised of α and β subunits critically important in regulating platelet functions. There are five integrins on the platelet surface: Two β3 integrins: αIIbβ3 (GP IIb/IIIa or CD41/61) which binds collagen and fibrinogen, αvβ3 which binds vitronectin (Table 1). Additionally, there are three β1 integrins: α2β1 which binds collagen, αvβ1 which binds fibronectin, and α6β1which binds laminin [14]. The most abundant integrin on platelet surfaces, αIIbβ3, has been extensively studied for its role in potentiating platelet activation [15]. Typically, αIIbβ3 is in an inactive (low affinity) state where it is less likely to bind fibrinogen even though there is a significant amount circulating in the blood. When platelets become activated due to exposure to damaged endothelium or to various agonists, αIIbβ3 switches to its active conformation (“inside-out signaling”). This change to the high affinity conformation allows for high-affinity binding to fibrinogen and initiation of downstream signaling events which mediate clot formation (“outside-in signaling”) [16]. 

The αIIbβ3 integrin plays specific roles in many different infections, including dengue fever, human immunodeficiency virus (HIV), and hantavirus infection indicating its significance in the immune system [17,18]. With respect to dengue fever, there is an apparent increase and subsequent activation in CD61 positive cells, along with other pre-cursor hematopoietic progenitor cells, released from the bone marrow during the acute stage of infection [17]. In fact, this increase in hematopoiesis and subsequent forcing of progenitor cells from the bone marrow as a result of increased cytokines, such as interleukin (IL)-3, IL-7, and granulocyte macrophage colony stimulating factor (GM-CSF) [19] may be the mechanism behind the “break bone” symptom, a commonly cited symptom of dengue where patients report severe bone pain [17]. Dengue virus (DENV) is then taken up by the CD61+ cells (in this case αIIbβ3 expressing platelets), where they can be protected from immune attack. Monocytes subsequently phagocytose these platelets. However, the macrophages are unable to kill the virus as it is protected within the confines of the platelet and is subsequently not neutralized by the immune cell [17,20]. Patients with dengue fever often see a decline in the measured amount of the virus’s RNA within the blood between days three and five of the infection, which is likely a result of its intracellular protection in CD61+ platelets during this time [17]. αIIbβ3 integrins are also considered receptors for hantaviruses which cause hemorrhagic fever with renal syndrome and hantavirus pulmonary syndrome. It has been reported that quiescent platelets can bind to hantaviruses in a β3 dependent manner [21]. This can be a basis of hantavirus induced thrombocytopenia as well as altered endothelial cell properties. 

Furthermore, with respect to HIV, αIIbβ3 has been shown to cross react with antibodies against the gp120 antigen on retrovirus surfaces [18], indicating a structural similarity between αIIbβ3 and viral antigen. In HIV infected patients, this cross reactivity causes immune thrombocytopenic purpura (ITP), as antibodies attack both retroviruses and integrin-expressing platelets, leading to a low platelet count [18]. Additionally, several bacteria such as *Staphylococcus aureus*, *Staphylococcus epidermidis*, and *Streptococcus pyogenes* produce fibrinogen/fibronectin binding proteins. As fibrinogen is a ligand for αIIbβ3, these proteins form a bridge between the surface of the pathogen and αIIbβ3 present on quiescent platelets leading to platelet activation [22,23]. Moreover, surface proteins from *Streptococcus gordonii*, which is directly recognized by αIIbβ3, induces platelet aggregation [24]. Such manipulation of the coagulation system may help pathogens to gain advantage over the host immune system [22,23]. Finally, during an *E. coli* infection, thromboinflammation is upregulated by simultaneous activation of FcγRIIA receptors and the αIIbβ3 integrin on platelets [25]. Bacterial killing of *E. coli* by platelets requires both FcγRIIA and platelet factor 4 (PF4) in order for destruction to occur [26]. Although the αIIbβ3 integrin is not directly involved in immune response against *E. coli*, it is a necessary component for the activation of platelet FcγRIIA and can lead to an upregulation of thromboinflammation. Indeed, while the expression of selectins and integrins on its surfaces are meant for immune assistance, many infections, such as dengue fever, HIV, *E. coli*, *Staphylococcus* and *Streptococcus* infections, can use these structures for their own advantages and produce adverse effects as a result. 

### 2.2. The Role of αvβ3 Integrin in Immunity

αvβ3 is another type of integrin expressed on platelet surface which participates in inflammation [14]. It plays a role in a myriad of processes such as osteoclast bone resorption, tumor metastasis, and perhaps most importantly, angiogenesis [27]. Its binding to tumor cells forces the flipping of the “angiogenic switch,” causing the expression of several important angiogenic proteins and cytokines in the platelet’s ECM, including fibroblast growth factor-2 (FGF-2), matrix metalloproteinase MMP-2, platelet-derived growth factor (PDGF), and vascular endothelium growth factor (VEGF) [27]. When these factors are expressed at damaged sites through binding to αvβ3, platelets spur endothelial growth and vessel formation, which can carry leukocytes and other factors to fight infections, sites of inflammation, or towards tumor sites through these new vessels [27,28]. 

### 2.3. The Role of P-Selectin in Inflammation and Infection

One of the most widely studied adhesion receptors in platelets is P-selectin, a 140 kDa glycoprotein composed of a 120 amino acid lectin domain, an epidermal growth factor domain, nine complement receptor repeats, a membrane spanning domain, and a cytoplasmic tail [29]. Following platelet activation, P-selectin is translocated to the platelet surface from alpha granules and mediates rolling of platelets on activated endothelial cells. Platelet P-selectin also binds to Mac-1 and PSGL-1 on neutrophils during platelet aggregation in sites of infection or inflammation to form platelet-neutrophil complexes (PNCs) [30]. Neutrophils in PNCs display a greater level of activation than those uncoupled from platelets, expressing more superoxide and phagocytosing more bacteria than their counterparts [31]. In fact, terms like “thromboinflammation” and “immunohemostasis” have gained traction in recent years in order to properly describe the interactive role of PNC’s in the immune response [26]. Upon activation via a variety of infection or inflammation mechanisms, platelets can change expression of their surface receptors to attach to other platelets, leukocytes, or extracellular pathogens, forming a complex that can help sequester and eliminate the pathogen [26]. 

While P-selectin’s role in platelet aggregation is well established, it is also thought to contribute to immunity in cases of cutaneous contact hypersensitivity [32]. Studies on the initiation of the complement system from the platelet surface via P-selectin have shown that it relies on the formation of the C3 convertase complex [33]. The complement component C1q also seems to mediate a moderate up-regulation of P-selectin on platelet surfaces presenting P-selectin’s role in immunity beyond adhesion [34]. Moreover, P-selectin mediated aggregation is also observed during *Helicobacter pylori* infection [35]. However, there is a possibility that P-selectin expressed by endothelial cells is participating in this process. Recently, other adhesion molecules such as E-cadherin are also being explored for their contribution to platelet aggregation but their roles in immunity needs to be identified [36].

## 3. Pattern Recognition Receptors (PRR) Assist Platelets in Direct Recognition of Pathogens

Pattern recognition receptors (PRRs) are germ line encoded receptors expressed by most innate immune cells. They recognize conserved structures among microorganisms called pathogen associated molecular patterns (PAMPs) [37]. Activation of these receptors leads to induction of different signaling pathways, mainly resulting in the initiation of inflammatory responses [38]. Some of these receptors may also be utilized by pathogens to enter the host cells. Different classes of PRR families including toll like receptors (TLRs), C-type lectin like receptors (CTLRs), and nucleotide binding oligomerization domain (NOD)-like receptors (NLRs) have been found to be expressed by platelets. The presence of these different types of immune receptors implicates platelets as significant contributors to the immune response [39]. 

### 3.1. Toll Like Receptors Are a Major Family of Receptors Contributing to the Role of Platelets in Infections

One of the major families of PRRs studied in context of platelet activation is the TLRs [40]. These are transmembrane proteins with a cytoplasmic toll/interleukin-1 (IL-1) receptor (TIR) domain, transmembrane domain, and an extracellular domain comprised of leucine-rich motifs [41]. TLRs are at the forefront of the innate immune response being some of the main drivers of inflammation following microbial infection [42]. Several studies in the early 2000s demonstrated the presence of functional TLRs in and on platelets including TLR1, TLR2, TLR4, TLR6, TLR7, TLR8, and TLR9, which become activated in response to PAMPs (Table 2) [39,41,42,43]. 

TLR4 is one of the most studied receptors in inflammation and is a sensor of lipopolysaccharide (LPS) molecules from Gram-negative bacteria. It is reported to be present at high levels on platelets [43]. In 2005, Andonegui et al. showed that platelets express TLR4, which is activated in response to LPS purified from *E. coli* and can result in thrombocytopenia as well as accumulation of platelets in the lungs in a neutrophil dependent manner [41]. It was quickly followed by another study with similar results showing that functional TLR4 is required for LPS-induced thrombocytopenia [44]. It has been observed that septic patients with thrombocytopenia had higher levels of TLR4 expressed on the platelet surface and more sCD40L in circulation, indicating increased activation levels [45]. As bacterial LPS can induce rapid thrombocytopenia, the literature suggests that activation of platelets through TLR4 can be a major mechanism for such thrombocytopenia in sepsis [46,47]. In contrast, a recent study has shown that purified LPS does not activate platelets and only whole live enterohemorrhagic *E. coli* O111 can induce platelet activation [48]. The lack of platelet activation in response to LPS purified from *E. coli* O157, other strains of *E. coli*, *P. aeruginosa*, and *K. pneumoniae* have been reported in other studies as well, questioning the relevance of platelet TLR4 in these infections [49,50,51]. These discrepancies might be a result of the ability of platelet TLR4 to distinguish various isoforms of LPS [52] along with differences in experimental setups such as the use of different bacterial strains, variations in the method of platelet preparation and LPS purification, and differences in the ratio of platelets to bacteria. 

Additionally, there is evidence to suggest that simultaneous addition of low concentrations of platelet agonists such as thrombin along with LPS from *E. coli* O111 is essential to induce TLR4 dependent platelet aggregation [53]. Moreover, the maximal oxidative phosphorylation capacity of platelets was found to increase as a response to LPS from *K. pneumoniae* and an increased level of ROS production by platelets was observed in response to LPS from *E. coli*, which is blocked by TLR4 inhibitors [49]. This implies that TLR4 can modulate the metabolism of platelets even in absence of classical platelet activation. In addition to LPS, there are several endogenous as well as exogenous ligands, including fibronectin, fibrinogen, DENV NS1, mannan teichuronic acid from gram positive bacteria, which has been reported to activate TLR4 on innate immune cells and induce cytokine production [54,55,56,57]. Hence, it is likely that similar effects can be observed from these agonists on platelet TLR4, which needs to be further explored. 

TLR2 is another functional PRR in platelets and has roles in different bacterial as well as viral infections. Though the major ligands for TLR2 are lipoproteins, its ability to recognize other microbial components such as LPS, peptidoglycans, lipoteichoic acid, and zymosan originates from its ability to form heterodimers with TLR1 and TLR6 [56,57,58] One of the earliest studies about the functionality of platelet TLR2 was performed with *Rickettsia africae*, the causative agent of African tick bite fever [59]. It was shown that stimulation of platelet rich plasma (PRP) from humans by *R. africae* enhanced the levels of soluble CD40L (sCD40L), while antibodies against TLR2 could block this effect. Similar results were observed upon the stimulation of TLR2 in PRP by its synthetic ligand, Pam3Cys [59]. As activated platelets are considered to be the major source of sCD40L [60], it is likely that these effects were a result of TLR2 mediated platelet activation. Functional expression of TLR2 in platelets was confirmed when the activation of this receptor by its agonist, Pam3CSK4, induced platelet aggregation in a dose dependent manner [42]. Furthermore, there are several studies characterizing the role of TLR2 in infections caused by periodontopathogens such as *A. actinomycetemcomitans* and *P. gingivalis*, demonstrating TLR2 dependent upregulation of CD40L on the platelet surface [61,62]. A recent study also indicated that the increase in release of CD40L from the platelet surface was through TLR2 and NF-kB signaling [63]. Another study showed that TLR2 also participates in the infections mediated by human cytomegalovirus (HCMV). When TLR2 is inhibited or knocked out, HCMV mediated activation of platelets is blocked [64]. Interestingly, this activation does not induce platelet aggregation, indicating that HCMV interaction with platelets is not responsible for its prothrombotic effects [64].

The available literature also presents platelet TLR2 as a major player in facilitating the interaction of platelets with other immune cells, mainly neutrophils [62]. When platelets from TLR2 knockout mice were incubated with wild type neutrophils, mice platelets are unable to form aggregates with neutrophils in response to periodontopathogens [62]. In an in vitro study, the incubation of human platelets, stimulated using TLR2 agonist Pam3CSK4, with human neutrophils with were found to enhance neutrophil phagocytosis as indicated by the internalization of FITC conjugated latex beads [65]. On the contrary, markers of neutrophil activation such as CD11b were found to decrease upon co-culture with stimulated platelets [65]. The opposing effects of increasing phagocytosis while inhibiting neutrophil activation indicates that platelet TLRs can play an immunomodulatory role [65].

Studies of platelet endosomal TLRs are very recent and limited. In 2014, it was found that TLR3, responsible for the recognition of double stranded RNA, is present on platelet surface as well as in intracellular compartments [66]. Through an in vitro experiment, it was shown that a synthetic analog of double stranded RNA, Poly I:C, can lead to increased calcium concentration, CD62P expression, and TLR3 expression on human platelets [66]. However, whether these results are due to direct interaction between TLR3 and poly I:C is not well understood. Nevertheless, it demonstrates that platelets express a functional TLR3 and might be involved in innate immune responses [66]. In the same year, TLR7, another receptor involved in recognition of viral RNA, was found to be expressed by human as well as mouse platelets [67]. TLR7 is activated by single stranded RNA and its stimulation in platelets is implicated in innate immunity through cross communication with neutrophils [67]. Interestingly, activation of platelet TLR7 was found to result in an increase in the percentage of platelets associated with granulocytes even after inhibition of neutrophil TLR7, implying that platelets are the initiators of the association [67]. Under in vitro conditions, an increase in the levels of myeloperoxidase released by human neutrophils was observed in response to platelet TLR7 activation [68]. Additionally, platelet TLR7 is involved in mediating the release of C3 in response to influenza infections, which can further induce neutrophil extracellular trap (NET) formation [68]. As elevated coronary NET burden is a predictor of myocardial infarct size, it is possible that the release of web-like structures from neutrophils during NET formation gives rise to a highly prothrombotic condition by providing a scaffold for platelets and may be responsible for the increased risk of myocardial infarction (MI) observed in influenza [68,69]. However, direct interaction of platelets with virus via TLR7 is not yet confirmed. Recently, the co-localization of HIV pesudovirions with TLR7-containing compartments after endocytosis by platelets was also demonstrated [68,70]. TLR8 is another receptor which is conventionally considered to be an endosomal receptor for single stranded RNA [71]. However, recent studies show that TLR8 can be involved in recognizing a number of other PAMPs [72]. In the case of platelet, high levels of expression of TLR8 mRNA was observed in thrombin activated platelets treated with chitin from *C. albicans* [73]. However, the exact role of such increased TLR8 is unknown. As TLR7 and TLR8 are found to have a significantly lower level of expression than other TLRs in platelets with a higher level found in females than males [74], the extent to which these receptors contribute to platelet function needs to be further explored.

There is also some evidence to show that the surface expression of platelet TLR9, thought to be localized in a distinct electron-dense intracellular compartment within a platelet called T granule, is upregulated in response to synthetic unmethylated Type C CpG oligonucleotides [76]. Similar increased expression of TLR9 was also found in platelets from patients of acute coronary syndrome (ACS) in comparison to healthy individuals. In response to oligodeoxynucleotide (ODN) 2006, another agonist for TLR9, the ACS platelets also exhibit increased expression of activation markers such as CD62P [78]. TLR9 knockouts have significantly decreased levels of CD62P surface expression, along with nearly 50% reduction in sequestration of type C CpG [76]. A non-canonical ligand for TLR9: carboxy alkylpyrrole protein adducts (CAP) has also been recognized. It acts in a TLR9 dependent manner to increase the aggregation and activation of platelets in vitro [77]. The activation of platelets by thrombin receptor activating peptide (TRAP) was also found to increase the expression levels of platelet TLR5 and TLR9 [49]. Furthermore, the presence of viruses such as HIV-1 in intraplatelet structures indicate that intracellular TLR9 can play a role in mediating viral responses following endocytosis of the virus by platelets. Indeed, a recent study involving platelets from endocytosis deficient mice showed that endocytic trafficking is essential for stimulation of TLR7 and TLR9 by HIV-1 as well as its agonists such as CpG ODN2395 [70]. The recognition of viral particles by these TLRs is followed by downstream signals involving Akt, SNAP-23 and interleukin 1 receptor-associated kinase 4 (IRAK) lead to platelet leukocyte aggregate formation and platelet activation [70]. Additional roles of platelet TLRs in other bacterial and viral infections are subjects of further investigation. 

### 3.2. Several Other Families of PRRs also Contribute to the Role of Platelets in Immunity

Another family of PRRs described in platelets are the CTLRs which detect carbohydrate structures derived from pathogens through their conserved carbohydrate recognition domains [79]. Several studies have demonstrated the expression of dendritic cell specific ICAM grabbing non integrin (DC-SIGN) and C-type lectin-like receptor 2 (CLEC-2) in platelets and their role in viral infections [80,81]. In 2005, CLEC-2 was identified as a novel receptor present on platelets which can lead to platelet activation in response to a snake venom toxin called rhodocytin [80]. It was quickly followed by other studies which demonstrated the functions of platelet CLEC-2 in HIV infections [81,82,83]. The expression of DC-SIGN by platelets was also demonstrated in the same year by Boukour et al. [81]. It was discovered that both DC-SIGN and CLEC-2 can be involved in the capture and internalization of HIV-1 by platelets [82]. The inhibition of these lectin type receptors was found to significantly decrease the association of HIV-1 with platelets. Similar reports of the ability of platelets to internalize HIV-1 through their lectin receptors, platelet CTLRs have also been studied in context of DENV infection. It was shown that NS1 can directly bind platelets and induce platelet activation as well as apoptosis in a dose dependent manner [84,85]. When DENV is incubated with platelets and washed, surface bound virus as well as internalized virus were obtained, confirming the direct binding of platelets to DENV [84]. Upon the blocking of DC-SIGN by monoclonal antibody, the effects of DENV on platelets were attenuated significantly [84]. In another study, the platelets from DENV infected patients were observed to have lower levels of expression of DC-SIGN [86]. Such alteration in DC-SIGN levels might be a way to prevent uncontrolled activation of the innate immune system mediated by platelets [86]. A recent study reported that the use of anti-CLEC2 monoclonal antibody was able to suppress the activation of platelets induced by DENV [87]. It was also found that such activation via CLEC-2 was causing the release of extracellular vesicles from platelets which increased the formation of NETs and cytokine production [87]. As mentioned previously, platelet TLR4 is also implicated in DENV infections. It is possible that different strains of DENV work through different receptors or there can be several ways for DENV to interact with platelets (as mentioned previously in Section 2.1). Finally, by binding to its in vivo ligand podoplanin, platelet CLEC-2 is also thought to be associated with driving thrombosis after *Salmonella typhimurium* infection [88]. While further evidence will help to confirm the role of these lectin receptors in platelets, the available literature supports the role of platelet CTLRs in pathogenesis of infections. 

The literature regarding other families of PRRs such as NOD-like receptors in platelets is lacking. NOD-like receptors are cytoplasmic receptors with a C-terminal leucine-rich repeat (LRR) domain, a NOD in the center and variable N-terminal domain involved in interaction with other proteins [89]. NOD1 and NOD2 are the two main types of NOD receptors with one and two caspase recruitment domains, respectively [90]. In 2015, NOD2 was shown to be expressed at both the RNA and protein levels in human as well as mouse platelets [90]. However, muramyldipeptide (MDP) did not induce any platelet activation even at high concentrations, but interestingly, potentiating effects were observed on thrombin mediated activation even at low concentrations of MDP [90]. These effects were absent in platelets of NOD2 knockout mice. The study also shed some light on the role of platelet NOD2 in thrombus formation and hemostasis. When PRP from mice was treated with platelet-poor plasma (PPP) from humans with bacteremia, the levels of platelet aggregation induced by collagen and thrombin was lower in the PRP from NOD2 deficient mice [90]. This can mean that there is some role of NOD2 in platelet hyperreactivity and prothrombotic state observed in infection. An attempt to understand its mechanism was also made where the receptor interacting protein 2 (RIP2)/MAPK pathway was found to be involved in NOD2 mediated platelet activation [90]. The expression of other classes of PRRs including retinoic acid-inducible gene I (RIG-I), which is involved in viral recognition, is confirmed in megakaryocytes during its response to type 1 interferons, but its presence in platelets is not known [38]. Nevertheless, it is certain that platelets have diverse mechanisms to participate in immune responses to viral infections irrespective of a direct or indirect interaction between PRRs and viruses [38]. 

## 4. The Only Fc Receptor on the Platelet Surface, FcγRIIA, Binds to Opsonized Pathogens and Bacterial Surface Proteins

FcγRIIA, also referred to as CD32a, is the only Fc receptor known to be expressed on human platelets [91]. Platelets were shown to bind IgG immune complexes through a Fc receptor which was later characterized as FcγRIIA. This receptor is an activating FcγR that has a low affinity for monomeric IgG molecules, but high avidity for IgG-containing immune complexes (ICs). The structure of FcγRIIA is composed of two Ig-like domains in their extracellular portion, a membrane spanning region, and an immunoreceptor tyrosine-based activation motif (ITAM). The second region of the extracellular Ig-like domain mediates the binding to IgG. The Ig-like domain can also interact with IgG-opsonized pathogens. In the cytoplasmic domain of FcγRIIA, the ITAM region, a conserved signaling motif with the consensus sequence Yxx(I/L)x(6-12)Yxx(I/L), where x is any amino acid, is responsible for mediating intracellular signaling (reviewed in [92]). 

In the context of platelet functions mediated by FcγRIIA, most of the studies are conducted by incubating different agonists, mainly aggregated IgGs, crosslinked IV.3 monoclonal antibody (mAb), and activating antiplatelet antibodies, with isolated human platelets [93]. In the case of aggregated IgGs, which are implicated in the pathogenesis of autoimmune diseases such as systemic lupus erythmatosus (SLE) and anti-phospholipid syndrome (APS), interaction with FcγRIIA results in platelet activation, α-granule release, dense granule release, and microvesicle formation [94,95,96]. Multiple studies have shown that a humanized anti-FcγRIIA antibody (VIB9600) might function as a therapeutic for autoimmune diseases, including systemic lupus erythematosus, rheumatoid arthritis, and vasculitis [97,98]. FcγRIIA has also been shown to have a role in inducing pro-inflammatory cytokines active in Kawasaki disease and Grave’s disease [99,100]. Furthermore, FcγRIIA participates in immune mediated heparin induced thrombocytopenia (HIT), which is a complication associated with the anticoagulant drug heparin [101]. PF4, released by platelets, binds to heparin such that an immunogenic PF4-heparin complex is formed. This leads to the generation of antibodies targeted against the complex, called HIT antibodies, which can activate platelets through FcγRIIA, resulting in thrombocytopenia [101]. Moreover, variations are observed in the platelet response to HIT antibodies depending on the density of FcγRIIA on the platelet surface and its polymorphisms [102]. Diseases like idiopathic thrombocytopenic purpura (ITP) are characterized by the presence of antiplatelet antibodies which can also crosslink FcγRIIA either on adjacent platelets or the same platelet [93] and lead to platelet aggregation, increase in cytosolic calcium concentration, and granule secretion. 

FcγRIIA also responds to diverse IgG-opsonized pathogens [93]. Several studies show that the surface proteins from pathogens such as *Staphylococcus aureus*, *Streptococcus oralis*, *Streptococcus sanguinis*, and *E. coli* bind to IgG leading to crosslinking of FcγRIIA and platelet aggregation [51,103,104,105]. Platelet FcγRIIA also plays a role in viral infection. H1N1 can form immune complexes with IgG, leading to platelet activation [106]. Due to the antibody dependent mechanisms of FcγRIIA and its role in heparin induced thrombocytopenia (HIT), it is thought that sepsis associated thrombocytopenia may be a result of increased surface expression FcγRIIA in platelets during infections [86,107]. However, a study on DENV infection of platelets has shown that the surface levels of FcγRIIA actually decrease as the severity of infection increases [86]. As the receptor is also thought to provide the entry for virus into platelets, it is possible that such lower levels of the receptor are a consequence of the immune system attempting to diminish virus mediated thrombocytopenia. Additionally, the same receptor may respond differently during bacterial and viral infections. For instance, previous studies show that *E. coli* stimulates platelet aggregation via FcγRIIA in a Syk and Src dependent manner [51] while other bacteria, specifically *Staphylococcus* and *Streptococcus* strains, rely on secondary mediators to induce platelet aggregation [103]. Hence, elucidating the functions and signaling of FcγRIIA in platelets is important in order to understand the role of platelets in infectious diseases.

## 5. Complement Receptors on Platelets Are Important in Potentiating Immune Responses

The complement system is an innate defense system comprised of acute phase proteins critical for host defense and known to be involved in thrombosis [108]. By binding to their individual receptors, these proteins can result in activation of signaling pathways, initiating the complement cascade which directly leads to lysis of opsonized pathogens [109]. Human platelets have been shown to contain complement proteins [110] and to recognize and bind complement proteins through complement receptors. The types of complement receptors found on platelets include cC1qR, gC1qR, C3aR, C5aR, CR2, and P-selectin [110] which enable platelets to respond to pathogens.

### 5.1. Different Receptors on Platelets Recognize Complement Component 1q Leading to Platelet Aggregation

Complement components 1q (C1q) is a 460-kDa protein consisting of six heterotrimeric collagen-like triple helices that associate in their N-terminal half that diverge into individual “stems” with each ending in a C-terminal heterotrimeric globular domain [111]. One of the earliest evidences for the presence of specific binding sites for C1q on platelets was provided by Peerschke and Ghebrehiwet in 1987 [112]. This was followed by several other studies identifying cC1qR and gC1qR as the platelet receptors responsible for recognizing C1q [109,113,114,115]. The recognition by cC1qR revolves around the N-terminal collagen-like domain of C1q [116]. gC1qR interacts with the C-terminal globular domain on C1q (gC1q) [113]. Both domains may participate in platelet activation as binding of C1q to platelets has shown to induce platelet aggregation and P-selectin expression [117,118].

gC1qR, also referred to as p33, is a single chain, multiligand protein with an apparent molecular mass of 33 kDa [119]. It has been shown to have a wide cellular distribution which includes platelets and endothelial cells [119]. It was shown that platelets activated by shear stress can activate the classical pathway through gC1qR [114]. A moderate upregulation of P-selectin on platelet surfaces also seems to be initiated by gC1qR binding C1q [34]. Additionally, gC1qR has been shown to interact with microbial organisms [110]. There is evidence that platelet gC1qR plays a role in the recognition of *Staphylococcus aureus* through *S. aureus* protein A [120]. A few studies have also indicated gC1qR mediated aggregation of platelets in response to *S. sanguis* in a FcγRIIA dependent manner [121]. Moreover, an immune complex involving C1q and M1 protein from *S. pyogenes* was found to be associated with complement activation on platelets and increased phagocytosis by monocytes, but the role of platelet C1q receptors was not proven [122]. In multiple other studies, the endospore of *Bacillus cereus*, a Gram-positive bacteria, and viral antigens such as human immunodeficiency virus (HIV) and Epstein Barr virus (EBV) were also shown to have a binding site for gC1qR [123,124,125]. However, these pathogens have not been proven to bind with the receptor on platelets and require further investigation.

Finally, C1q can potentiate platelet activation, aggregation, and granule secretion by aggregated IgG [126]. Such enhanced responses were absent in the presence of polyclonal antibody targeted against cC1qR, implying the functionality of complement receptors in platelets [126]. However, very few studies have been conducted examining the direct role of cC1qR in infections. Given the crucial role of the complement system in responding to pathogens, further studies into the role of C1qR in platelets could lead to a better understanding of the immune functions of platelets. 

### 5.2. G protein Coupled Receptors, C3aR and C5aR, Are Expressed on Platelets and Bind to Products of Complement Activation

C3aR is a G protein coupled receptor (GPCR) that is predominantly expressed on immune cells, adipocytes, epithelial cells, liver, kidneys, heart, as well as the brain [127]. It binds to a 77 amino acid peptide called C3a, an anaphylatoxin, generated by complement activation [127]. The literature regarding the role of C3aR in platelets is mainly focused on thrombosis. The interaction of C3a with C3aR in platelets can activate the small GTPase Rap1b, promote αIIbβ3, which can both contribute to coagulation and hemostasis [128,129]. C3aR also regulates the specific steps of thrombus formation including platelet adhesion, spreading, and Ca^2+^ [130]. 

Similar to C3aR, C5aR is a GPCR which binds with high affinity to a 74 amino acid containing anaphylatoxin: C5a [130]. Structurally, C5aR is made up of seven transmembrane regions connected by loops on both the extracellular and intracellular sides. The interaction of C5a with C5aR involves at least two sites on C5aR and leads to several immune responses such as myeloid cells migration and vasodilation [131]. An investigation of the role of platelets with patients suffering from coronary artery disease (CAD) shows that expression of C5aR on platelets to be correlated with platelet activation [132]. There are few studies which show possible links between C5aR with diseases like asthma and Alzheimer’s disease [131]. While it is known that lymphocytes play a role in potential therapeutic treatments of these diseases, with the recent discovery of C5aR on platelets, it is unknown if platelets play a role too. Further studies may lead to findings demonstrating the role of activated C5aR in platelet aggregation, like C3aR.

Both C3aR and C5aR respond to products of complement activation and evidence to support the role of these receptors in actively binding to pathogens is lacking. Nevertheless, the role of platelets in infections can be implied based on the ability of complement system to induce platelet aggregation through C3aR. With recent studies demonstrating the protective role of C3aR and C5aR in infections caused by *Neisseria meningitis* and *Listeria monocytogenes* respectively, along with evidence to support thrombosis triggered by promotion of C3a in *E. coli* O157:H7 infections [133,134,135], further studies are required to assert the specific role of platelet C3aR and C5aR in these infections. 

### 5.3. Complement Receptor 2 Contributes to Response of Platelets During Viral Infection

Complement receptor 2 (CR2) is a type I transmembrane protein found on mature B cells, peripheral T cells, thymocytes, basophils, mast cells, keratinocytes, epithelial cells, and platelets [136]. It is approximately 145 kDa with 15–16 short consensus repeats, a 28 amino acid transmembrane domain, and a 34 amino acid cytoplasmic tail [33]. There are four classes of ligands characterized as binding partners of CR2: iC3b, C3d, C3dg, and the gp350/220 protein present in the viral coat of Epstein Barr virus [137]. Though the cross linking of these receptors is shown to cause platelet activation and aggregation [113], enough studies have not been conducted to elucidate whether CR2 on platelets responds to either iC3b or C3d. Nevertheless, platelets are shown to interact with the Epstein-Barr virus (EBV) via CR2 [115,138]. Platelets binding to EBV causes release of TGF-β, which is a strong immunosuppressive cytokine [138]. Such release of TGF-β can regulate to immune cell proliferation and differentiation and tissue fibrosis, emphasizing the role of platelets in the immune response against EBV [139]. Additionally, finding a link between C3d bound pathogens and CR2 may provide another way in which platelets bind to and interact with pathogens.

## 6. The Role of Scavenger Receptors in Platelet Mediated Immunity is not Well Understood

Scavenger receptors (SRs) are an incredibly large group of membrane bound receptors predominantly expressed on myeloid cells. They are divided into ten classes (A-J) based on their structure [140]. While the structure and primary sequence of the different classes of scavenger receptors can vary greatly, they are all united by their ability to bind similar ligands such as pathogens, apoptotic cells, proteoglycans, ferritin, and carbohydrates [141]. The functions of SRs are as complex as they are diverse. Although primarily thought to facilitate the recognition and removal of non-self and altered-self molecules, it has been shown more recently that SRs also have roles in angiogenesis, atherogenesis, lipid metabolism, and immune surveillance [140]. Initially classified on macrophages for the purpose of recognizing and clearing low-density lipoprotein (LDL), SRs have also been discovered to be expressed on platelets. While platelets express a variety of different scavenger receptors, two scavenger receptors from Class B are found to have substantial effects on resting platelets, namely CD36 (also known as SR-B2) and SR-B1 [142]. 

CD36 is a transmembrane scavenger receptor that recognizes a variety of different ligands such as thrombospondin-1, fatty acids, oxidized LDL (oxLDL), microbial diacylglycerides, and others [142]. It is expressed by many cell types and has a variety of functions, but in platelets, the binding of ligands to CD36 initiates signaling causing platelets to enter into a hyperreactive state [143]. Clinically, CD36 activation during hyperlipidemia and high blood LDL levels increases the likelihood of atherosclerosis and coronary artery disease [144]. Interestingly, CD36 deficiencies do not appear to increase the risk of bleeding disorders [145], and deficiencies in CD36 or pharmacological blocking of JNK kinases lead to less thrombotic events in hyperlipidemic mice [143]. While multiple studies present CD36 and its downstream effectors as potential therapeutic targets for antithrombotic treatments, very few studies have explored the role of platelet CD36 in infections. Due to its ability to act as a coreceptor for TLR2 along with its role as a phagocytic receptor in macrophages and dendritic cells, it can be expected that CD36 plays similar roles in platelets. CD36 from macrophages has been implicated in immune responses against *S. aureus* and *S. pneumoniae* infections [146,147]. However, the platelet specific role of CD36 in these infections is unknown [146]. Additionally, platelet activation stimulated by *Plasmodium falciparum* during cerebral malaria has been shown to involve CD36. *P. falciparum* infected red blood cells (RBCs), can engage CD36 on platelets, leading to their activation and release of platelet factor 4 (PF4) to promote inflammation [148]. As CD36 in RBCs binds directly to membrane proteins of *P. falciparum*, direct binding of platelet CD36 to the parasite may be possible as well.

Another scavenger receptor B1 family member found in platelets is SR-B1, which is structurally similar to CD36 and is primarily expressed on macrophages, hepatocytes, and dendritic cells [146]. While SR-B1 primarily functions to mediate the selective transport of cholesteryl esters from HDL in hepatic cells, it also plays a role in platelet aggregation [144]. Activation of SR-B1 with high-density lipoprotein requires the recruitment of the scaffolding protein PDZK, leading to the activation of Src, PI3K, protein kinase B (Akt) or MAPK, and eNOS [149]. SR-B1 activation has been shown to modulate the inhibition of platelet aggregation in vitro [144], and reduced expression of SR-B1, PDZK1, and Akt1 have been shown to increase the risk of spontaneous or diet induced coronary artery atherosclerosis and myocardial infarction in mice [150]. Like CD36, the role of SR-B1 in infections is not well studied. In hepatocytes, SR-B1 has been shown to support the entry of hepatitis C virus and *P. falciparum*, while its expression in HeLa cells has shown to mediate the uptake of bacteria such as *L. monocytogenes* and *S. typhimurium* [151]. However, in vivo evidence for such roles and its relevance in platelets is unavailable. Additionally, SR-B1 deficient mice have been shown to have poor disease outcomes in response to *Klebsiella pneumoniae* and *Mycobacterium tuberculosis* infections [152,153]. Therefore, it can be inferred that although the potential clinical aspects of platelet SR-B1 have not yet been scientifically explored, it could play a significant role in mediating the immune functions of platelets.

In platelets, activation of SRs CD36 and SR-B1 play important roles in permitting and inhibiting platelet activation, respectively. Though CD36 and SR-B1 are important modulators of platelet activity as they are expressed on quiescent platelets, it is important to note that there are many more SRs expressed in platelets such as lectin-like oxidized low-density lipoprotein receptor-1 (LOX-1), SR-A, and CD68 especially in an activation dependent manner [142]. LOX-1 is an SR expressed on endothelial cells, macrophages, smooth muscle cells, and platelets that primarily binds oxLDL, apoptotic cells, bacteria, and activated platelets [142]. LOX-1′s expression in platelets is activation dependent [154] and appears to assist platelet aggregation after activation; as blocking of LOX-1 inhibited ADP initiated platelet-activation in vitro [155]. Furthermore, recent studies have investigated its role in *Aspergillus fumigatus* and *Escherichia coli* infections through studies in LOX-1 deficient mice [156,157]. However, the identities and functions of these SRs are not well understood in platelets. Hence, it is certain that platelet SRs can modulate their activity and further studies are required to understand the role of SRs in infections as they could prove to play a critical role in not only platelet function, but also clinical disease and treatments.

## 7. Platelets Express Several Other Receptors Involved in Infection and Inflammation

GPIb-IX-V: In addition to the categories of receptors described above, there are several other receptors, some of which are exclusive to platelets and contribute to their immune functions. GPIb-IX-V is one such receptor specifically expressed by platelets and binds to von Willebrand factor (vWF) [158]. This complex is comprised of four glycoprotein subunits where GPIbα and GPIbβ are connected by a disulphide bridge to form GPIb, while GPIX and GPV are linked in a noncovalent manner [159]. In addition to participating in platelet aggregation and activation as a complex, its individual components can participate in different immunological responses. For instance, GPIbα is thought to mediate adhesion of bacteria such as *Streptococcus sanguis* and *Streptococcus gordinii* to platelets through direct recognition of bacterial proteins [160,161]. *S. aureus* and *Helicobacter pylori* are also capable of producing proteins which bind to vWF and interact with GPIbβ [162,163]. Moreover, the inhibition of GPIbα has also been shown to promote pulmonary metastasis in a P-selectin dependent manner [164]. 

GPVI: GPVI is another platelet specific receptor which plays a crucial role in hemostasis by binding to collagen and initiating the early stages of platelet aggregation [165]. Beyond hemostasis, deletion of platelet GPVI is associated with increased growth of *Klebsiella pneumoniae* in lungs [166]. Additionally, when GPVI is stimulated in human platelets using its agonist cross-linked collagen related peptide (CRP-XL), platelet-leukocyte complexes were increased, and leukocytes showed improved ability to phagocytose *Klebsiella pneumoniae* [166]. GPVI also interacts with hepatitis C virus (HCV) through its ectodomains though it is not clear if this interaction results in endocytosis of the virus [167]. Furthermore, GPVI, in combination with the thromboxane A2 receptor (TP), participates in promoting synthesis of proinflammatory mediators from macrophages during zymosan induced inflammation [168]. The levels of surface expression of these glycoproteins on the platelet surface are regulated by a family of metalloproteinases called A Disintegrin And Metalloproteinase (ADAM). Specifically, ADAM10 and ADAM17 are present on surface of resting platelets and facilitate the cleavage of GPVI and GPIbα, respectively [165]. In addition to its regulatory role in receptor shedding, platelet derived ADAM10 and ADAM17 also have roles in modulating immune responses [165]. They participate in the cleavage of NKG2D ligands expressed by tumor cells, hindering the recognition of these tumor cells by NK cells. A higher level of expression of ADAM10 and ADAM17 was found in platelets from lung cancer patients than healthy controls, indicating the possibility that these receptors can facilitate immune evasion for tumor cells. [169]. Furthermore, deletion of ADAM10 on platelets as well as myeloid lineage cells has been shown to protect mice from sepsis induced by *Staphylococcus aureus.* In vitro, the alpha toxin produced by *S. aureus* was shown to impair platelet aggregation by increasing the activity of ADAM10, which leads to proteolysis of GPVI receptor [170]. Additionally, lower levels of ADAM10 in platelets is also associated with patients of Alzheimer’s disease [171]. ADAM10 has also been shown to facilitate HIV-1 replication in macrophages and CD4+ cells such that similar roles in platelets may be possible [172]. 

Siglec Receptors: Siglec receptors are also expressed by platelets and have the potential to mediate their responses to infection and inflammation. These are a family of immunoglobulin-like lectins which bind to sialic acid based ligands from host cells as well as pathogens and are expressed by a variety of cells including dendritic cells, macrophages, and neutrophils [173]. They are also involved in negatively regulating the pathways initiated by TLRs in immune cells [174]. Significant levels of Siglec7 and Siglec9 have been confirmed in the activated platelet [40,175]. Though the stimulation of Siglec 7 by ganglioside, its ligand, does not lead to platelet aggregation, its cross linking has been shown to induce platelet apoptosis [175]. As Siglec 7 has been shown to have inhibitory effects in natural killer cells [176], it is possible that platelet apoptosis is a way to negatively regulate the inflammatory responses mediated by platelets. Furthermore, the sialic acid groups from capsule of Group B *Streptococci*, have also been shown to inhibit platelet activation by engaging Siglec 9 on the platelet surface [40]. Such suppressive effects on platelets are thought to provide Group B *Streptococci* with resistance against platelet mediated killing [40]. In natural killer cells, Siglec 7 and Siglec 9 are known to participate in a number of infections including HIV-1, hepatitis B, hepatitis C and also facilitate immune escape for tumor cells [177]. Therefore, it is highly likely that Siglecs expressed by platelets have similar roles and further studies can provide valuable insights into their roles in platelet-mediated immunity.

5HT2A: Few studies also present 5-hydroxytryptamine 2A receptor (commonly known as serotonin or 5HT2A receptor) as a mediator of platelet responses to infections. 5HT2A receptor is a GPCR widely known to regulate platelet physiology [178]. Due to the significant role of serotonin in regulation of the central nervous system, a major portion of the studies involving 5HT2A is focused on psychological disorders and few studies have reported the immunoregulatory functions of serotonin [178]. Recently, it was shown that serotonin generated from mast cells during dengue infections acts on platelets through 5HT2A receptor, leading to their activation and aggregation with eventual thrombocytopenia [178]. Moreover, this is associated with a positive feedback loop as platelets store large concentrations of serotonin in their dense granules which is released upon platelet activation [178]. This also places platelets in a central position to deliver serotonin to other inflammatory effector cells including macrophages, microglia, mast cells, neutrophils, and dendritic cells [178]. The use of drugs such as ketanserin, which inhibits 5HT2A receptors, were also shown to protect mice from thrombocytopenia during DENV infection [178]. Such results present 5HT2A as a promising candidate for treatment of dengue and further studies may illustrate the significance of platelet 5HT2A receptor in other viral infections as well. 

## 8. Discussion and Conclusions

**Figure 1:**
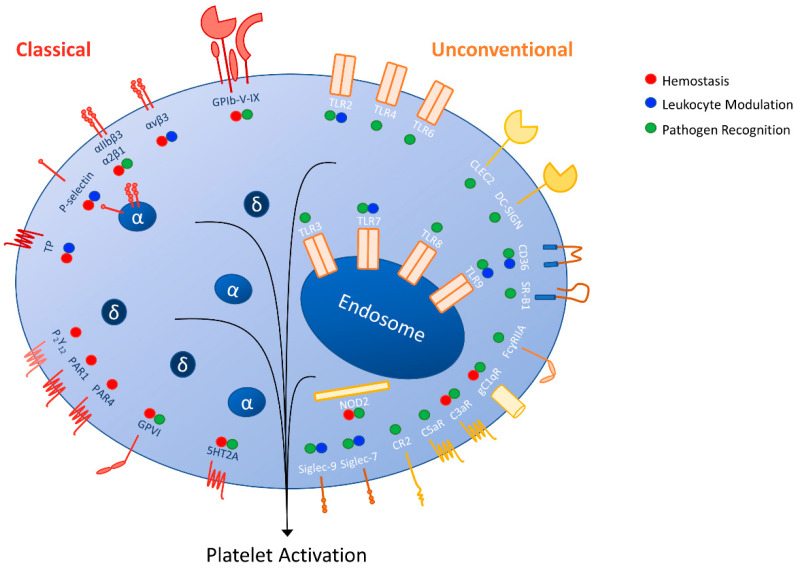
Classical and unconventional platelet receptors and their contributions to immunity and hemostasis.

Platelets express their “classical” receptors involved in normal hemostatic function, and, as described in this review, they also express receptors not originally thought to be present in platelets, or “unconventional” receptors. Whether they are considered classical or unconventional, we have described how both types of receptors contribute to platelet responses during infection and inflammation (summarized in Figure 1). The actions of platelets mediated by these receptors can be beneficial for the host and sometimes for the pathogen, depending on the specific consequences of receptor activation. Recent studies have led to a better understanding of the role of platelet receptors in infections, though questions have been raised about the functionality of conventional signal transduction pathways in platelets. As platelets are anucleate, their signal transduction is likely to differ than other nucleated immune cells and further studies are required to elucidate the mechanism of action of immune receptors in platelets. 

Platelet-specific receptors are also being recognized as good targets for prevention or treatment of thrombotic complications associated with infections (Table 3). For example, inhibitor of Bruton’s tyrosine kinase, ibrutinib, was recently shown to block CLEC-2 mediated platelet activation in human under in vitro conditions and may be used to prevent thrombo-inflammatory conditions associated with DENV and HIV [179]. Use of 5HT2A antagonists, such as ketanserin, are another platelet-targeted therapy that has been shown to prevent DENV-associated thrombocytopenia in mice [180]. Furthermore, the use of antibodies against FcγRIIA has been shown to suppress thrombocytopenia and inflammatory responses mediated by immune complexes [181]. Similarly, blocking Siglec 9 is a potential therapy to reduce severity during *Streptococcal* infections by reducing the resistance to platelet mediated killing [182,183]. Additionally, modulation of actions mediated by platelet receptors can also affect the responses from other immune cells. The recognition of the same pathogen by different categories of receptors may also indicate that a single pathogen can utilize multiple receptors to coordinate its virulence mechanisms in platelets. Knowledge about such coordinated signaling pathways in platelets and their roles in regulating immune responses will lead to development of targeted therapies for treatment and prevention of severe bacterial as well as viral infections. Hence, additional studies are required to assert the safety and specificity of these receptor targeting drugs in humans under in vivo conditions. Further clinical development of platelet receptors as therapeutic targets can decrease the overall morbidity and mortality rates associated with different types of infections. 

## Figures and Tables

**Table 1:** Types of β3 integrins on platelets with a known role in immunity.

Integrin Subtype	CD Marker	Ligands	Result	References
αIIbβ3	CD41/61	Collagen; Fibrinogen; DENV; Hantavirus; Fibrinogen binding proteins (FBPs) from bacteria; *Streptococcus gordonii* surface proteins	Platelet activation; Platelet aggregation; Virus recognition and internalization; Upregulation of thromboinflammation	[14,16,17,18,21,22,23,24,26]
αvβ3	CD49e/CD61	Vitronectin	Platelet activation Platelet aggregation Leukocyte migration Endothelial growth Angiogenesis Tumor metastasis Osteoclast bone resorption	[14,27,28]

**Table 2:** Types of TLRs expressed by platelets and their role in infections.

TLRs	Agonists	Functions	References
TLR2	Bacterial Lipoproteins; Pam3CSK4; HCMV; *P. gingivalis*	sCD40L upregulation and release; Platelet aggregation; Platelet activation; Platelet-neutrophil aggregates; Increased phagocytosis by neutrophils	[58,59,61,62,63,64,65,75]
TLR3	Double stranded RNA; Poly I:C	P-selectin expression; Increased calcium concentration	[66]
TLR4	Gram-negative bacterial LPS	Platelet activation; Platelet accumulation; sCD40L release; Increased oxidative phosphorylation capacity; ROS production; Infection-induced thrombocytopenia	[41,43,44,45,46,47,48,49]
TLR7	Single stranded RNA	Increased association with granulocytes; Upregulation of NET formation	[67,69]
TLR9	Synthetic unmethylated Type C CpG oligonucleotides; CAP adducts	Platelet activation and P-selectin expression; Platelet aggregation Platelet leukocyte aggregates	[70,76,77,78]

**Table 3:** Platelet receptors as potential therapeutic targets in infection and inflammation.

Platelet Receptors	Pathogen Recognized	Adverse Consequences Facilitated by Receptor Activation	Potential Receptor Targeting Therapeutics	References
αIIbβ3	HIV DENV Hantavirus *S. aureus* *E. coli*	ITP; Virus induced thrombocytopenia; Altered endothelial cell properties; Platelet aggregation; Thromboinflammation	Abiciximab Eptifibatibe Tirofiban	[21,23,38,183]
DC-SIGN	HIV DENV	Viral entry and replication; NET formation	Dextran Isomaltooligosaccharides Quinoxalinones	[184,185]
CLEC-2	DENV HIV *Salmonella typhimurium*	Viral entry and replication; Thrombosis	Cobalt hematoporphyrin Ibrutinib	[179,186]
FcγRIIA	*S. aureus* *E. coli* DENV	Sepsis associated thrombocytopenia HIT SLE APS Kawasaki disease	Anti-FcγRIIA antibodies (IV.3 & VIB9600)	[180]
CR2	EBV	TGF-b release	Soluble recombinant CR2 proteins	[187]
Siglec 9	Group B *Streptococci*	Resistance against platelet mediated killing	Anti-Siglec 9 antibodies	[182,183]
5HT2A	DENV	Thrombocytopenia	Ketanserin	[178]
